# Effects of Lavender Essential Oil Inhalation on the Welfare and Meat Quality of Fattening Heavy Pigs Intended for Parma Ham Production

**DOI:** 10.3390/ani13182967

**Published:** 2023-09-20

**Authors:** Eleonora Nannoni, Giovanna Martelli, Maurizio Scozzoli, Simona Belperio, Giovanni Buonaiuto, Niccolò Ian Vannetti, Eleonora Truzzi, Enrico Rossi, Stefania Benvenuti, Luca Sardi

**Affiliations:** 1 Department of Veterinary Medical Sciences (DIMEVET), Via Tolara di Sopra 50, 40064 Ozzano Emilia, Italy; eleonora.nannoni2@unibo.it (E.N.); simona.belperio2@unibo.it (S.B.); giovanni.buonaiuto@unibo.it (G.B.); niccoloian.vannetti2@unibo.it (N.I.V.); luca.sardi@unibo.it (L.S.); 2 Italian Society for Research on Essential Oils (Società Italiana per la Ricerca sugli Oli Essenziali—SIROE), Viale Regina Elena, 299, 00161 Roma, Italy; 3 Department of Life Sciences, Via G. Campi 103, 41125 Modena, Italy; eleonora.truzzi@unimore.it (E.T.); enrico.rossi@unimore.it (E.R.); stefania.benvenuti@unimore.it (S.B.)

**Keywords:** animal welfare, pig welfare, phytoextracts, meat quality, carcass traits, skin lesions, lavender oil, inhalation administration, aggressive behavior

## Abstract

**Simple Summary:**

This study aimed to investigate lavender essential oil aromatherapy as a calming phytoextract to improve the welfare of fattening Italian heavy pigs (intended for Parma ham production) and its possible effects on pig meat. Three pig groups were formed: one raised in commercial conditions, one receiving lavender oil inhalation administration once a day and one receiving lavender oil twice a day. We observed no effects of lavender on carcass or blood stress indicators, and only minor effects on meat quality, not affecting the subsequent dry curing processing. No residues were found in lean or fat tissue. With respect to welfare, animals treated once a day showed less severe tail lesions (indicating a lower level of frustration and damaging behaviors) compared to the other groups. Unexpectedly, animals treated twice a day showed a more severe degree of lesions on the body compared to the other groups (possibly indicating increased agonistic behaviors). Although from these conflicting results it was not possible to conclude on the ability of the product to improve the level of animal welfare, further studies are needed to investigate the potential effects on pig behavior and the optimization (frequency and modality) of the administration of vaporized lavender essential oil.

**Abstract:**

We assessed the effects of inhalation administration of lavender essential oil (LEO) either once (L1) or twice (L2) a day on animal welfare indicators, carcass and meat quality of Italian heavy pigs. Pigs (*n* = 108) were allotted to three experimental groups (control -C-, L1 and L2) and lavender was administered, via a vaporizer device, to the treated groups during the entire fattening–finishing period (79–160 kg BW). Tail lesion severity was reduced in L1 at the end of the trial compared to the other groups (*p* < 0.05). Body lesion counts, however, were higher in L2 than in C (*p* < 0.05), resulting in a more severe overall damage classification (*p* < 0.01). At slaughter, no differences were observed in carcass traits or blood stress indicators, only minor differences were observed in meat quality, and no LEO residual was found in fat or lean tissues, highlighting the preserved suitability of thighs for the dry curing process. While it was not possible to conclude on the ability to improve animal welfare of vaporized LEO in this production phase, the absence of adverse effects on meat quality and the discrepancies observed regarding the body lesions in L1 and L2 make further studies on behavioral aspects and the method of administration (route, frequency) of the product desirable.

## 1. Introduction

The use of phytoextracts and active botanical ingredients in animal production has received considerable interest in recent years, mainly due to increased efforts to reduce both the environmental burden of drug use and antimicrobial resistance. Among the various uses, these extracts have been proposed as feed ingredients or water supplements for their nutraceutical, probiotic or immunomodulating effect, or for their influence on the final product quality [1].

Lavender (*Lavandula angustifolia* Mill.) essential oil (LEO) is a calming phytoextract that can be administered either orally or by inhalation. In laboratory animals such as gerbils [2] and rats [3], LEO inhalation resulted in anxiolytic effects that were in some cases similar to those obtained with drugs (benzodiazepines). Its use under commercial farm conditions has therefore the potential to reduce stress and anxiety in pigs, increasing their calmness level and promoting better performance and animal welfare.

Studies on the use of calming phytoextracts in pigs are limited and mainly refer to mixed plant extracts (e.g., rosemary, sage, lavender, valerian, passiflora, etc.) administered orally and during specific stressful events (e.g., transportation). For example, a commercial herbal product containing *Valeriana officinalis* and *Passiflora incarnata* administered in drinking water before transportation had antianxiety effects (smaller increase in selected heart variables) in young pigs (24 kg BW) [4]. The same product delivered in feed to growers (16 to 24 weeks of age) determined slightly increased BW and reduced hair cortisol at the end of the trial [5]. Similarly, Pastorelli et al. [6] fed *Passiflora incarnata* powder extract to piglets and hypothesized a calming and anti-anxiety effect of this plant extract determining a slight improvement in wellbeing (lower skin lesions, higher termographically assessed skin temperature).

Specific studies on LEO administration to pigs by inhalation or diffusion in the environment are limited. In an early study from Bradshaw et al. [7], lavender straw was provided as bedding to pigs during transportation, resulting in pigs being more active but showing lower signs of motion sickness compared to wheat straw bedding. More recently, Direksin et al. [8] suggested that smelling LEO for 4 h after transportation was not effective in preventing aggressive behavior, but it apparently allowed an earlier establishment of hierarchy compared to the unexposed group. The authors argued that by reducing motion sickness, LEO may have allowed pigs to adapt earlier to the new environment. Lastly, a recent work by Crone et al. [9] tested the use of LEO aroma as an environmental enrichment during transportation. Their results showed that pigs stood more and mounted each other more during transportation (likely in an attempt to reach and explore the lavender-infused sachets hanging from the sides of the trailer), which resulted in a similar level of lesions on the body across treatments, and therefore, no benefits of lavender extract aromatherapy during transport were reported. However, the authors argued that aroma dispersal as the truck was moving may have limited the possibility of detecting the effects of the lavender-infused enrichment.

Overall, as shown by the reviewed studies, lavender administration in relation to a stressful event (during or immediately after transportation) did not show consistent effects on animal welfare. In particular, unexpected results were found in some cases, with lavender administration (either as straw or as extract) increasing animals’ overall activity level during transport [7,9] and even aggression levels immediately after transportation [8]. Under these circumstances, LEO was nonetheless suggested as a means to promote animal adaptation to the new environment (either by reducing motion sickness or by settling hierarchy earlier) rather than as a method to promote calmness.

The present study tested the hypothesis that the inhalation administration of LEO to pigs for an extended period of time (i.e., during the entire finishing period) and when animals are not subjected to acute stressors may have beneficial effects on animal welfare by increasing calmness level and reducing aggressive behaviors. Long-term positive effects on animal welfare and adaptability could reflect also on growth parameters and on meat and carcass quality, a particularly important aspect when animals are raised under specific production rules [10] and their meat and carcass must comply with strict quality requirements in order to be suitable for the dry curing process.

With respect to the effects of LEO on meat quality, to the best of our knowledge, no studies are available on pigs. Studies on broilers demonstrated a positive impact on growth parameters of lavender powder in feed (400 ppm), suggesting its use as an alternative to antibiotic growth promoters. In this species, LEO did not affect carcass traits but improved meat quality (lower cooking loss and lipid peroxidation) [11]. Similar effects, together with a reduction in pathogens and an increase in probiotic bacteria in the gut microflora, were observed after LEO administration to broilers in drinking water (0.4 mL/L) [12].

So far, no studies investigated the possible presence of LEO residuals in the meat of treated animals. This assessment is particularly important within certified food schemes, since residuals may determine an odor/taste which could potentially affect the quality of the final product. In the present study, the HS-SPME (headspace solid-phase microextraction) technique coupled with gas chromatography analysis was chosen in order to verify the absence of any residues of LEO in the animals’ tissues [13,14] after inhalation administration and the preserved suitability of the raw thighs for the dry-curing process. In recent years, the most frequently used analytical techniques for the extraction and concentration of volatile compounds are those based on headspace (HS) analysis. This represents a reliable tool for the analysis of volatile organic compounds and eliminates most drawbacks to extracting organics, including high cost and excessive preparation time. In particular, SPME is a simple and fast modern tool used to characterize the volatile fraction of medicinal plants and foods. In biological materials, SPME has been successfully applied to the characterization and analysis of the volatile compounds from plasma and biological fluids. In this study, the HS-SPME technique combined with gas chromatography coupled with a flame ionization detector (GC-FID) was applied to study the volatile compounds in animal tissues after the administration of LEO [15].

The overall aim of the present study was therefore to assess, under common farming conditions for Italian heavy pigs intended for the production of Parma ham, the effects of the environmental diffusion (either once or twice a day) of LEO. The variables measured included welfare and health indicators (growth traits, skin and tail lesions, blood indicators), as well as carcass, meat and fat quality in order both to monitor potential welfare effects and to exclude alterations in meat quality traits.

## 2. Materials and Methods

The trial was carried out in the facilities of the Department of Veterinary Medical Sciences (DIMEVET) of the University of Bologna, Italy. The experiment did not include any invasive procedure in vivo, and therefore, the research project was authorized as an observational study by the Ethical committee of the University of Bologna, with protocol number 3610, date of approval 10 January 2023. The rearing phase was carried out in full compliance with the EU legislation on pig protection [16], and animals were inspected at least once a day.

### 2.1. Pigs, Housing and Feeding

One hundred and eight crossbred (Goland × Large White) barrows with undocked tails were used. Pigs were individually identified with ear tags upon arrival and homogeneously allotted to three experimental groups on the basis of their initial body weight (BW). The average BW and age at the beginning of the trial were approximately 79 kg and 154 days, respectively. Pigs were kept in collective pens on a partially slatted floor, and no mixing occurred during the experimental period. Each pen was equipped with a nipple drinker, a collective stainless-steel feeder and an environmental enrichment tool (soft wooden logs hanging from a wall). Pigs were located in temperature- and humidity-controlled rooms equipped with a forced-air ventilation system (RH was set at 65% and T was set at 23 °C). Lighting was artificial and was supplied by neon tubes (from 7:00 a.m. to 7:00 p.m.).

Animals were fed using two commercial feed formulations (first phase up to 110 kg BW: 3195 kcal DE/kg DM, CP 14.50% DM; second phase from 110 kg BW to the end of the trial: 3210 kcal DE/kg DM, CP 14.20% DM). Liquid feed (water/meal ratio 3:1) was offered twice a day (at 8:00 a.m. and 3:00 p.m.), with meal rationed at 9.5% of the metabolic BW (BW^0.75^). The daily rations were adjusted every 2 weeks on the basis of the expected growth and of intermediate weighings, up to a maximum of 3.4 kg of meal/head/day.

Animals were kept under the same experimental conditions until they reached the slaughtering body weight (160 Kg ± 10%) requested by Parma Ham production rules [10].

### 2.2. Experimental Groups

Each experimental group included 6 replications of 6 pigs each, for a total of 36 animals per group. Groups were raised in different rooms of the experimental barn (one room per group) to avoid possible confounding effects due to unintended lavender exposure. The groups were defined as follows:

- Control group (**C**) was kept at the standard experimental conditions described above;
- Once-a-day lavender group (**L1**) was kept in the same experimental conditions, with the only difference being that a solution containing 1% lavender (*Lavandula angustifoli a* Mill.) essential oil (LEO) was vaporized in the room once a day (at 7:00 a.m.) for 10 min (approximately 200 mL of solution used at each vaporization session) The mixture and the vaporizer were prototypes custom-made for this experimental trial.
- Twice-a-day lavender group (**L2**) was kept in the same experimental conditions as C, but LEO vaporization took place twice a day (at 7:00 a.m. and 12:00 p.m.) for 10 min each time (approximately 200 mL of solution used at each vaporization session).

The times at which vaporization sessions took place were selected trying to avoid possible associations between LEO vaporization and feeding time or other daily routine activities (inspections, room cleaning, etc.), in order to avoid possible confounding effects due to pigs anticipating what would happen close to the vaporization sessions.

### 2.3. Tail and Skin Lesions Assessment on Farm

The presence of skin and tail biting lesions was assessed according to the Welfare Quality^®^ protocol for growing and finishing pigs [17]. Lesions were scored at the beginning and at the end of the trial. The number of lesions on one side of each animal was counted for each body region (ears, front, middle, thighs and legs) and used for classification: each region was scored as a (up to 4 lesions), b (5–10 lesions) or c (11–15 lesions). Each pig was then scored using a 0-to-2 scale, where 0 corresponded to a pig having the full body classified as ‘a’, 1 to a pig having any body region scored as ‘b’ and/or a maximum of one region scored as ‘c’; and 2 to a pig having at least two body regions or more classified as ‘c’, or at least one body region with more than 15 lesions. Tail biting was assessed according to the following scale: 0 (intact tail, no evidence of tail biting), 1 (superficial biting but no evidence of fresh blood or swelling) and 2 (fresh blood, evidence of swelling or infection, or tissue missing with the formation of a crust).

### 2.4. Carcass and Meat Quality Traits

Pigs were shipped at an average weight of 169 kg, according to the production rules for Parma Ham production [10]. They were transported and slaughtered according to the EU legislation [18,19]. Animals were transported for approximately 1 h and delivered to a commercial abattoir, where they were slaughtered after a 15 h fast, immediately after unloading (no lairage time). During transport, treatment groups were kept separated (no mixing). Animals were stunned (head-only electrical stunning) and bled, and carcasses were processed using conventional practices. A blood sample was collected at exsanguination and analyzed as described in Section 2.4.

At the slaughter plant, carcass weight and the weight of the main carcass cuts (thigh, and loin) were recorded; lean meat percentage and back-fat thickness were measured by Fat-o-Meater (FOM-SFK, Copenhagen, Degnmark). The yield of the carcass and of the main cuts were later calculated based on carcass weight. Carcass lesions were visually assessed with the same method described for on-farm lesion assessment, with the only difference being that only the visible parts of the carcass were scored (shoulder, flank, thigh, legs).

Using a portable pH meter (model 250 A, Orion Research, Boston, MA, USA), pH was measured in the *Longissimus thorachis* (LT) and *Semimembranosus* (SM) muscles at 45 min post-mortem (pH45′) and in the SM at 24 h post-mortem (pH24 h). At 24 h post-mortem, instrumental color (Minolta CR-400 Chromameter Minolta Camera, Osaka, Japan, D65 illuminant, color space L*a*b*) was measured in the *Longissimus lumborum* (LL) and SM muscles, and samples of LL muscle were taken in order to determine drip loss and cooking loss according to Honikel [20]. Shear Force was measured on 6 cores from the cooked samples using an Instron Universal Testing Machine, model 1011 (Instron Ltd., High Wycombe, UK) fitted with a Warner–Bratzler (WB) device at a cross-head speed of 200 mm/min. Meat and subcutaneous fat samples were also collected and analyzed as described in Section 2.5.

### 2.5. Blood Samples Collection and Analysis

At exsanguination, a blood sample was collected from a subsample of 24 randomly selected pigs for each experimental group (*n* = 72). Blood was collected in lithium heparin tubes and immediately stored at +4 °C to be transferred to the laboratory of the Department of Veterinary Medicine of the University of Bologna. Blood tubes were then centrifuged (at 2000× *g* for 20 min), and plasma was separated and stored at −20 °C, pending subsequent analysis for cortisol, CK, and aldolase.

Cortisol was used in this study as an indicator of acute pre-slaughter stress (e.g., during handling, loading, transport and/or restraint) [21]. Creatine kinase (CK) was chosen as a subacute (8–48 h) indicator of intense physical activity (including aggressive behaviors and activity during the last on-farm stages and on the truck) [22,23,24]. Lastly, aldolase was taken as a long-term indicator of muscle damage (48–72 h) also correlated to meat quality traits [25,26,27,28].

The quantitative determination of serum cortisol was made using a commercial Elisa kit (orb566639, Biorbyt Ltd., Cambridge, UK) and is expressed as ng/mL.

Creatine kinase and aldolase concentrations (both expressed as U/L) were measured using two commercially available kits (CK Nac Liquid and Aldolase, Sentinel Diagnostics, Milan, Italy), and serum CK and aldolase concentrations were determined with a spectrophotometer (Ultrospec 3000, Pharmacia Biotech, Milan, Italy). The intra-assay CV was 5.61%, 4.21% and 4.98% for plasma aldolase, CK and cortisol, respectively.

### 2.6. LEO Residuals in Subucutaneous Fat and Muscle

A sample of lean meat (LL muscle) and a sample of subcutaneous fat (from the thigh region) were taken at the slaughter plant. Samples were immediately refrigerated and transferred to the DIMEVET laboratories, where they were finely minced and stored in plastic tubes that were sealed under vacuum and preserved at −20 °C pending the subsequent analysis, which was carried out within 1 month in the laboratories of Department of Life Sciences (University of Modena and Reggio Emilia).

Chemicals used in this analysis were linalool analytical standard (Sigma-Aldrich, Milan, Italy, CAS No. 78-70-6) and paraffin oil (Sigma-Aldrich, Milan, Italy, CAS No. 8012-95-1).

HS-SPME was performed using a manual holder (Supelco, Milan, Italy) and a stableflex 50/30 μm divinylbenzene-carboxen-polydimethylsiloxane (DVB-CAR-PDMS; Supelco, Milan, Italy) fiber. The coating was 1 cm long. Before GC analysis, the fiber was conditioned in the injector of the GC system, according to the instructions provided by the manufacturer.

About 1.5 g of the sample was weighed in a 15 mL glass vial (O.D. × H 21 mm × 70 mm), and 1 mL of paraffine was added. The vial was closed with a PTFE/silicone septum cap and rolled up with parafilm to avoid any gas leak. The sample was shredded in a magnetic stirrer for 5 min to destroy the tissues and cells of lean meat and subcutaneous fat. The sample was incubated in an ultrasonic bath for 15 min at 50 °C to saturate the headspace.

After the saturation time, the SPME fiber was exposed for 15 min, at the same temperature. Then, the fiber was manually transferred to the GC inlet for the desorption step in splitless mode for 5 min at 250 °C.

The GC analyses were carried out on a 7820 A gas chromatograph (Agilent Technologies, Milan, Italy) coupled with a flame ionization detector (FID). Compounds were separated on an Agilent Technologies HP-5 cross-linked poly-5% diphenyl–95% dimethyl polysiloxane (30 m × 0.32 mm i.d., 0.25 μm film thickness) capillary column.

The column temperature was initially set at 70 °C for 2 min, then increased at a rate of 2 °C/min up to 100 °C and held for 2 min, then ramped to 250 °C at 5 °C/min rate and held for 2 min. Helium was used as the carrier gas, at a flow rate of 1 mL/min. The injector and detector temperatures were set at 250 and 300 °C, respectively. The analyses were performed in triplicate.

To evaluate linalool as a marker of LEO in animal tissues, the calibration curve was created by analyzing the different concentrations of linalool standard dissolved in the control samples. Paraffin was selected as a diluent for linalool to prepare the solutions at different concentrations. The paraffin was analyzed via HS-SPME, and no volatile compounds were detected in the chromatographic analysis.

The correlation of linalool concentration and areas of the GC peaks is y = 26 × x − 1.35, R^2^ = 0.972. The limit of determination (LOD) has been calculated for both pig samples (lard and muscle), and it is related to a linalool concentration of less than 0.25 nL/g.

### 2.7. Statistical Analysis

The software Statistica (StatSoft Inc., release 12, 2013) was used. The pen was tested as a random effect, but it led to no significant differences, and therefore, the model was simplified to include only the experimental group. Therefore, continuous data (carcass and meat quality traits and blood indicators) were subjected to one-way ANOVA using the experimental group as the main factor. Significant differences were then analyzed by carrying out pairwise comparisons using the Bonferroni test. For non-parametric variables (lesion counts) the Kruskal–Wallis test was used (followed by multiple comparisons of mean ranks if needed). Class distributions (of skin and body lesions) were analyzed using the chi-squared statistics.

The pen was used as the experimental unit for growth parameters. The individual was taken to be the experimental unit for all the data collected. The significance level was set at *p* < 0.05 for all tests.

## 3. Results

During the on-farm trial and the journey to slaughter, no sanitary problem occurred that may have altered the trial results and interpretation. Animal carcasses were subjected to veterinary inspection at the slaughterhouse, and they were all judged to be fit for human consumption.

The growth parameters recorded during the on-farm trial (data not shown) did not show any significant difference among the experimental groups in any of the parameters considered (body weight, average daily gain, feed consumption, feed conversion ratio). In the fattening phase (80–160 kg BW), the average daily gain was 702 g, and the feed conversion ratio was 4.26.

Table 1 and Table 2 show the lesions observed on the body and tail of the animals. In particular, Table 1 shows the average lesion count per each area and the overall number of lesions on the body, while Table 2 shows the percentage distribution of animals in severity classes for tail and body lesions. With respect to lesion counts, as expected, no differences were observed at the beginning of the trial in any body region. In groups C and L1, the number of lesions was numerically lower at the end of the trial compared to the beginning (with the only exception of thigh lesions in L1), but the same did not happen in group L2. At the end of the trial, significant differences were observed among groups for thigh lesions and for the total number of lesions on the body, with L2 showing the greatest number of lesions for both types of lesions, significantly higher compared to C (*p* < 0.05 and *p* < 0.01, respectively). A significant difference was also observed across the three groups (*p* < 0.05) for flank lesions; however, the multiple comparisons test highlighted only a tendential difference (*p* < 0.1), with L2 group showing a greater number of flank lesions than C.

Similarly, as concerns lesion severity classes, no significant difference across groups was observed in class distribution at the beginning of the trial. At the end of the trial, the increased number of lesions described above resulted in a significantly higher presence of mildly damaged animals in group L2 compared to both L1 (*p* < 0.01) and C (*p* < 0.05). With respect to tail lesions, L1 showed the best class distribution (less animals whose tail was classified as 1, i.e., moderately damaged) compared to both C and L2 (*p* < 0.01).

The lesions on the carcasses are summarized in Table 3. No statistically significant difference was observed among groups, and only a tendential difference (*p* < 0.1) was found in the shoulder region, with the L1 group having tendentially more lesions than C.

Table 4 shows slaughtering and carcass quality parameters (including the main cuts yield), and Table 5 summarizes meat quality parameters (pH, color, water holding capacity and tenderness). No differences were observed among groups with respect to carcass parameters, whereas minor differences were observed in meat quality. In particular, meat from L1 animals showed lower pH values compared to group C, and animals subjected to the lavender treatment (both L1 and L2) showed higher drip loss (i.e., lower WHC, Water Holding Capacity) than C animals (*p* < 0.05 for all differences).

Table 6 shows the analytical results of stress indicators in blood. The three groups did not show statistically significant differences.

With respect to LEO residuals in the sampled tissues, all the samples of subcutaneous fat and muscle from the treated animals did not exceed the LOD of linalool (limit area of 5.84 for the fat and 8.62 for the lean tissue, <0.25 nL/g).

## 4. Discussion

Growth parameters recorded on farm were in line with the national data for Italian heavy pigs intended for the production of dry-cured hams [29] and very similar to those obtained by Gallo et al. [30] on pigs of similar weight (90–170 kg BW).

As concerns the overall number of lesions across groups, it should be kept in mind that the assessment methods differ considerably, and it is quite hard to draw direct comparisons. From a general standpoint, the overall prevalence of lesions seems to be similar or lower compared to other studies. For example, Tavares et al. [31] scored both sides of the body and found a number of lesions comparable to the present study but a higher number of tail lesions. Calderón Díaz et al. [32] assessed lesions on farm during the growing phase and found that 45% of pigs had lesions on the body and 15% on the tail, percentages that appear to be similar to the present study. However, they did not evaluate lesion severity and provided no detailed description of how lesions were observed (distance, minimum lesion size), and therefore, the comparison must be interpreted with extreme caution. With respect to lesions on the carcasses, similarly to the present study, Čobanović et al. [33] assessed lesions at slaughter in heavy pigs (121–145 kg BW) using the Welfare Quality^®^ classification [17], and they found moderate carcass lesions (5–10 lesions on the body) in 26% and severe lesions (more than 15) in 55% of slaughtered pigs. In the present study, regardless of the experimental group and based on the visible areas, we observed 20% of moderately damaged carcasses and 5% of severely damaged carcasses (data not shown). Therefore, the carcass lesion distribution across severity classes observed by Čobanović et al. [33] seems to be more severe compared to the distribution obtained in the present study. However, it should be noted that, unfortunately, at slaughter, it was not possible to assess tail and ear lesions, and therefore, the carcass severity classification could not be reported in full.

At the end of the trial, group L2 presented more lesions on the entire body (and in particular a significant difference in the thigh region and a tendential difference in the flank area) than the control group, with group L1 being intermediate. The tails were less severely bitten in group L1 compared to both C and L2. Overall, none of the lavender treatments resulted in a major positive effect on body lesions, with the administration twice a day resulting in a slightly increased damage grade, as a consequence of a possible increase in aggression rate. The only positive effect was observed on tail damage, which was reduced by the administration once a day (L1). The negative effect of lavender administration twice a day (L2), however, disappeared at slaughter (i.e., after animals had been loaded, transported and unloaded), with carcasses showing no significant differences in lesion counts. This could imply a higher number of fights of C pigs during the pre-slaughter phases compared to L2.

In previous studies, an increased aggression level was recorded in pigs inhaling lavender for 4 h after transportation [8]. However, the authors argued that the increased aggression levels stimulated an earlier establishment of hierarchical stability after transportation. Increased activity levels during transportation were also reported in other studies, where either lavender straw [7] or a lavender-infused enrichments [9] were provided, although without negative consequences on animal welfare or lesion counts. Pastorelli et al. [6] tested a different calming phytoextract (*Passiflora incarnata* powder extract) administered in the diet and found a reduced number of lesions in postweaning piglets. To the best of our knowledge, no study investigated the effects of on-farm LEO administration on body lesions. The overall scarcity of evidence indicating an improvement in the welfare level of finishing pigs could be ascribed to two contrasting conditions: (1) as heavy pigs grow up, they spend a considerable part of their time lying (up to 87% [34]), and therefore, the calming effect of lavender might be difficult to observe, and (2) since they are subjected to increasing welfare challenges towards the end of the production cycle (high stocking density (kg/m^2^), hunger due to feed rationing, chronic exposure to a barren environment and slatted floors, etc. [35]), lavender may have had only a limited effect. It is also possible that pigs may have been bothered by the nebulization twice a day (by the noise of the machine or the aroma spreading in the room), or each administration may have woken up the animals and/or provoked some anticipation that could have determined a period of activation during and immediately after the nebulization(s) session(s). These hypotheses could be further investigated by studying the animals’ behavior through the analysis of both their overall time budget during the day and how they behave in correspondence with the nebulization sessions and immediately after. It is, however, worth remembering that administration times were chosen to minimize the risk of anticipation or association with other daily routine events (cleaning, feeding, inspection, etc.) to avoid causing anticipation/agitation, and therefore, this factor could reasonably be ruled out pending the behavioral analyses. It remains still uncertain whether providing more than two administrations per day to adult pigs and/or using a noiseless machinery may determine more positive effects or if animals would, however, increase their activity during or after the nebulization sessions.

Carcass quality was also in line with Italian heavy pigs’ traits recorded in other studies [36,37]. The absence of variations in carcass quality across the experimental groups agrees with the lack of differences detected in growth parameters. Most importantly, it also indicates, together with the minor effects detected on fresh meat quality, that the nebulization of lavender did not affect the main pork attributes. Therefore, the meat obtained is suitable for the production of dry-cured hams according to their production rules for carcass and green hams quality [10]. Also, as concerns the analysis of residues in carcasses, linalool (the predominant compound used as a reference marker for LEO in tissues) showed no detectable residues and, therefore, no possible alteration in meat quality attributes that could affect taste or odor. In fact, the limit of 0.25 nL/g indicates that below this threshold, the extraction and analysis system is unable to evaluate the presence of linalool with respect to the system adopted and the sensitivity of the instrumentation. Under these analytical conditions, linalool did not exceed the LOD limit in fat and muscle tissues of the treated animals. Considering the extremely low value, it is clear that there were no residues of linalool.

To the best of our knowledge, this is the first study investigating the effects of LEO on pork quality. The only other available study considering meat quality aspects was carried out in broilers and reported no effects on carcass traits and composition but also an improved meat quality due to lower cooking loss and lipid peroxidation [11]. In the present study, we observed that loin pH at 45′ post-mortem was significantly lower in the L1 group compared to the control group, with L2 being intermediate. As a consequence, the water holding capacity of L1 loins was reduced (i.e., resulted in higher drip loss values) compared to the control group. A difference was also observed for the L2 group, showing as well significantly higher drip loss values compared to C. It is well known that low pH and WHC in pork have been correlated to acute stressful situations prior to slaughter that may determine significant pork quality alteration such as PSE (pale, soft, exudative) meat. Our findings, however, did not highlight any change in meat color or tenderness [38]. The reduction in pH associated with a decrease in WHC may therefore have been due to stressing events occurring during the pre-slaughter phase. Since all pigs were handled and transported in the same way, it is possible that animals in the two lavender groups may have reacted worse to pre-slaughter conditions (handling, loading, transport, unloading and moving to the stunning chute) compared to the control animals. A similar effect was previously observed in studies on animals raised in high-welfare farms [39,40] and on pigs receiving environmental enrichment tools (a wood log vs. a metal chain) [41]. This hypothesis is corroborated by the fact that lesions at slaughter were similar across groups, and therefore, the observed differences in meat quality are more likely to depend on the conditions on farm rather than on an increased aggression level of LEO-treated pigs during transportation. On the other hand, considering that blood stress indicators assessed at exsanguination failed to highlight significant differences in stress levels or muscular damage across groups, it cannot be ruled out that meat quality differences may be due to an occasional finding. Based upon these considerations, it could be interesting to investigate with future studies whether maintaining the exposure to LEO also during transportation (and possibly lairage) or changing the administration route (oral administration) could result in beneficial effects without altering meat quality traits. It is noteworthy that previous studies carried out only during (or after) transportation failed to find a positive effect of lavender on animal behavior [7,8,9] so far.

## 5. Conclusions

This work aimed to assess the effects of on-farm lavender inhalation administration to fattening–finishing Italian heavy pigs intended for the production of typical dry-cured hams (Parma ham). Our results show that the administration of lavender essential oil once a day resulted in slightly improved animal welfare conditions (lower tail lesions), while administration twice a day did not show positive effects and led to increased lesions on the body. Blood parameters and carcass traits were unaffected by the treatment, and meat quality showed only minimal differences that did not affect its suitability for the dry-curing process. No linalool residues were detected in animal tissues. Notwithstanding the importance of providing adequate care and resources to farmed pigs (especially by adopting housing and management practices that go beyond the minimum legislation requirements), lavender administered once a day could represent an additional help within traditional food production schemes. However, before proposing it as a practical method to be adopted on farm, several gaps of knowledge remain to be filled, in particular as concerns dose, time and method of administration, and animal behavior during and after administration.

## Figures and Tables

**Table 1:** Skin lesions (count per each body region and overall number) of the three experimental groups (C = control, L1 = lavender nebulization once a day, L2 = lavender nebulization twice a day) assessed at the beginning and at the end of the trial. Different superscripts within the same row indicate significant differences (^a,b^ *p* < 0.05; ^A,B^ *p* < 0.01). Means with an asterisk within the same row are tendentially different (*p* < 0.1).

Treatment	C	L1	L2	SE ^1^	*p*-Value
**Animals, n.**	36	36	36		
**Ear**					
Beginning	1.91	2.37	1.53	0.138	0.0504
End	1.58	1.72	2.03	0.125	0.4869
**Shoulder**					
Beginning	3.26	2.89	2.83	0.223	0.4542
End	1.61	2.42	2.29	0.196	0.0539
**Flank**					
Beginning	1.35	1.00	0.81	0.121	0.6308
End	0.78 *	0.69	1.43 *	0.144	0.0280
**Thigh**					
Beginning	1.71	0.97	1.42	0.147	0.0648
End	1.28 ^b^	1.50 ^a,b^	2.17 ^a^	0.182	0.0346
**Legs**					
Beginning	0.29	0.49	0.19	0.0598	0.3263
End	0.083	0.36	0.23	0.0568	0.1171
**Total lesions on the body**					
Beginning	8.53	7.71	6.77	0.369	0.2865
End	5.33 ^B^	6.69 ^A,B^	8.14 ^A^	0.429	0.0015

^1^ Standard error.

**Table 2:** Skin and tail classification (expressed as a percentage) of the animals belonging to the three experimental groups (C = control, L1 = lavender nebulization once a day, L2 = lavender nebulization twice a day) assessed at the beginning and at the end of the trial. Different letters indicate significant differences in the class distribution between groups (A,B *p* < 0.01).

Treatment	C		L1		L2		*p*-Value
**Animals, n.**	**36**		**36**		36		
**Tail lesions severity (%)**							
*Beginning*							
Class 0 (intact)	50		64		58		0.800
Class 1 (mild or moderate)	44		33		36		
Class 2 (severe)	6		3		6		
*End*							
Class 0 (intact)	75	A	97	B	72	A	0.011
Class 1 (mild or moderate)	25		0		22		
Class 2 (severe)	0		3		6		
**Overall body lesions severity**							
*Beginning*							
Class 0 (low presence)	65		77		81		0.283
Class 1 (moderate presence)	35		23		19		
Class 2 (high presence)	0		0		0		
*End*							
Class 0 (low presence)	89	B	78	B	49	A	0.0003
Class 1 (moderate presence)	11		22		51		
Class 2 (high presence)	0		0		0		

**Table 3:** Skin lesions (count per each body region and overall number) of the three experimental groups (C = control, L1 = lavender nebulization once a day, L2 = lavender nebulization twice a day) assessed on the carcasses. Means with an asterisk within the same row are tendentially different between each other (*p* < 0.1).

Treatment	C	L1	L2	SE ^1^	*p*-Value
**Carcasses, n.**	36	36	36		
**Shoulder**	1.09 *	1.92 *	1.53	0.159	0.051
**Flank**	4.56	4.46	2.92	0.451	0.585
**Thigh**	1.38	1.00	1.38	0.137	0.441
**Legs**	0.00	0.00	0.077	0.017	0.105
**Total lesions on the body**	7.03	7.38	5.92	0.527	0.950

^1^ Standard error.

**Table 4:** Slaughtering parameters and carcass quality of the three experimental groups (C = control, L1 = lavender nebulization once a day, L2 = lavender nebulization twice a day).

Treatment	C	L1	L2	MSE ^1^	*p*-Value
**Carcasses. n.**	36	36	36		
**Live weight (LW), kg**	167.0	171.8	170.4	147.4	0.263
**Carcass weight (CW), kg**	141.0	144.2	142.9	111.1	0.400
**Carcass yield, %LW**	84.2	83.9	83.8	121.10	0.501
**Backfat thickness, mm**	30.3	31.4	28.4	38.31	0.134
**Lean yield (F-o-M), %**	52.03	51.45	52.78	8.30	0.150
**Loin weight, kg**	11.86	12.43	12.25	1.141	0.119
**Thigh weight (TW), kg**	16.26	16.83	16.63	1.470	0.201
**Loin yield, %CW**	17.11	17.38	17.21	1.485	0.702
**Thigh yield, %CW**	23.43	23.54	23.38	0.934	0.833
**Trimmed thigh weight, kg**	13.56	13.97	13.82	0.944	0.266
**Trimmed thigh weight loss, %TW**	16.60	16.98	16.92	1.743	0.499

^1^ Mean square error.

**Table 5:** Meat quality parameters of the three experimental groups (C = control, L1 = lavender nebulization once a day, L2 = lavender nebulization twice a day). Different superscripts within the same row indicate significant differences (^a,b^ *p* < 0.05).

Treatment	C	L1	L2	MSE ^1^	*p*-Value
**Samples, n.**	36	36	36		
**pH 45′ loin**	6.64 ^a^	6.49 ^b^	6.57 ^a,b^	0.047	0.041
**pH45′ thigh**	6.49	6.39	6.48	0.15	0.593
**pH 24 h thigh**	5.78	5.74	5.79	0.024	0.429
**Loin color (m** **. *Longissimus torachis*)**					
L *	45.70	43.92	45.48	39.16	0.529
a *	8.29	8.15	8.28	10.47	0.984
b *	3.68	3.64	3.62	3.08	0.992
**Hue**	24.51	24.22	24.80	122.09	0.983
**Chroma**	9.19	9.06	9.23	10.74	0.983
**Thigh color (m . *Semimembranosus)***					
L *	43.36	43.65	44.06	19.05	0.836
a *	9.26	8.72	8.72	5.55	0.600
b *	3.97	3.65	3.63	1.85	0.558
**Hue**	23.64	21.86	23.13	47.77	0.613
**Chroma**	10.14	9.53	9.50	6.08	0.536
**Drip loss, %**	1.66 ^b^	2.16 ^a^	2.23 ^a^	0.84	0.038
**Cooking loss, %**	28.00	27.62	29.71	73.38	0.366
**Warner–Bratzler shear force, kg/cm^2^**					
**Mean**	6.35	6.19	6.14	2.39	0.869
**Median**	6.25	6.17	6.17	2.36	0.976

^1^ Mean square error.

**Table 6:** Stress indicators (analyzed on blood collected at exsanguination) of the three experimental groups (C = control, L1 = lavender nebulization once a day, L2 = lavender nebulization twice a day).

Treatment	C	L1	L2	MSE ^1^	*p*-Value
**Samples. n.**	24	24	24		
**Cortisol, pg/mg**	16.43	16.46	15.35	56.43	0.771
**CK, pg/mg**	2009.98	2044.73	2016.90	1022647	0.989
**Aldolase**	49.54	44.33	48.05	564.2	0.661

^1^ Mean square error.

## Data Availability

Data reported in this study are available upon request to the authors.
